# Natural Materials Modified and Applied to the Detection of Drugs In Situ: Modification of Eggshell and Quantification of Oxytetracycline

**DOI:** 10.3390/s22155746

**Published:** 2022-08-01

**Authors:** Helena I. A. S. Gomes, M. Goreti F. Sales

**Affiliations:** 1 BioMark@ISEP/CEB—Centre of Biological Engineering/LABBELS, School of Engineering, Polytechnic Institute of Porto, Rua Dr. António Bernardino de Almeida, 4249-015 Porto, Portugal; hiasg@isep.ipp.pt; 2 BioMark@UC/CEB—Centre of Biological Engineering/LABBELS, Department of Chemical Engineering, Faculty of Sciences and Technology, University of Coimbra, Rua Sílvio Lima, Polo II, 3030-790 Coimbra, Portugal

**Keywords:** eggshell substrate, antibiotic, water, environment, aquaculture

## Abstract

This work describes a novel sensing system using eggshells as substrate for the first time, targeting the detection and semiquantitative determination of antibiotics in waters from aquaculture, enabling simple, inexpensive, and in situ drug monitoring. Eggshell was ground and the resulting powder was modified by adsorption of suitable reagents, and it takes a typical colour after contact with the antibiotic. The colour intensity is correlated with the concentration of the antibiotic. This novel approach was applied to oxytetracycline, one of the antibiotics commonly used in aquaculture. The chemical changes on the eggshell powder were evaluated and optimised to produce an intense colour change as a function of the concentration of the antibiotic. The colour changes were evaluated by visual comparison with images taken with a digital camera, applying an appropriate mathematical treatment to the colour coordinates of the HSL system used by Windows. The selectivity of the response was tested against other antibiotic drugs. The materials were also used in the analysis of a spiked environmental water sample. Overall, this work presents a rapid, inexpensive, simple and equipment-free method for screening and discrimination of tetracycline drugs in aquaculture. The method is a green approach by reusing eggshells and decreasing the level of contamination correlated to analytical methods, thus being a promising tool for local, rapid, and cost-effective antibiotic monitoring.

## 1. Introduction

Aquaculture production has increased over the decades to meet global demand for fish food, in a direction meant to increase food security and reduce poverty [1,2]. However, the increase in fish production has also been associated with contamination of aquatic environments with antibiotics used to prevent/treat fish diseases [3,4]. Antibiotics in the aquatic environment are also due to their overuse in clinical contexts. Because humans excrete large amounts of drugs or their biologically active derivatives through urine or faeces, antibiotics end in wastewater treatment plants where they are hardly eliminated [5]. The wastewater undergoes biological treatment with bacteria, and this is ineffective when the complete elimination of antibiotics is intended [6].

This uncontrolled contamination of water with antibiotics has global public health implications and is also associated with antibiotic resistance, which is critical to public health mostly considering that the antibiotics used in fish farming are the same as those used in clinical settings [3,7,8]. Urgent measures are required to control and reduce the levels of antibiotics disposed to waters. Having aquaculture as a target, the possibility of monitoring the levels of antibiotics more accurately to reduce the amount of antibiotics given to fish in aquaculture would be generally appreciated both by fish farmers and the public. This benefits producers through lower treatment costs and the environment and public health by reducing the spread of antibiotics in the water. However, strict control of antibiotic concentrations in the aquatic environment during fish treatment is necessary to keep antibiotic concentrations to a minimum.

While most routine methods are complex and expensive because they monitor a combination of drugs [9], monitoring a single antibiotic in water introduced in fish feed may be done using cost-effective and environmentally friendly methods that allow rapid on-site response so that immediate action can be taken. Sensor strips are the most suitable test format for this purpose, as dipping the stick into the water is sufficient to generate a signal, similar to pH paper strips. There are few antibiotic sensor strips described in the literature for antibiotic monitoring. One uses a PVC-based sensor strip for detecting fluoroquinolones within a suitable reagent entrapped in a plasticized PVC substrate [10]. Another work used a cellulose substrate chemically modified with a reagent that in the presence of tetracyclines yields a coloured product [11].

We propose here to apply this principle to a natural substrate that currently has no use and can be obtained in large quantities. It is eggshells, which are among the most abundant waste materials derived from food processing [12,13]. Eggshell has a porous structure that should be able to absorb chemical species that reacts with the target antibiotic to produce colour, acting as substrate for a possible test-strip. Using the eggshell support in a test-strip for the first time, as far as we know, a coloured product being formed on it should be visible. As the most used classes of antimicrobials in terms of frequency include tetracyclines [14], and oxytetracycline (OXY) was by far among the most used antibiotics in salmon in 2016 [15], this new analytical test was developed for OXY. OXY is an antibiotic of the tetracycline group, of low cost and broad spectrum, widely used for treating infections in humans and animals. Apart from the amount of OXY that reaches environmental waters directly, about 20% of OXY is eliminated from the body in its original form, meaning that vast amounts of the drug reach the environment, affecting aquatic microbial communities and driving forward antibiotic resistance. It is therefore of great relevance to monitor OXY in waters, not only as a contaminant, but also when it is being feed the fish in aquaculture facilities, aiming to decrease the amount of antibiotic administered locally. To this end, the novel method making use of eggshell residues considered that all chemical modifications made on the eggshell support were followed and evaluated by Raman spectroscopy and further optimised to obtain an intense colour change depending on the concentration of the antibiotic (optimization of complexation reactions between the metal and the antibiotic). The sensor strip was also validated by analyses of spiked water samples.

## 2. Experimental Section

### 2.1. Materials and Reagents

Deionized water <0.1 μS/cm was used for this work. The chemicals used were of proanalytical grade, which included iron (III) chloride 6-hydrate (Scharlau), copper (II) sulphate 5-hydrate (Panreac), tetracycline (TTC, Applichem), chloramphenicol (CAP, Fluka), OXY (Fluka), sulfadiazine (SDZ, Fluka), and sodium chloride (NaCl, Panreac). Eggshells were household waste eggshells and commercial sodium hypochlorite was used for eggshell hygiene.

### 2.2. Apparatus

An ultrasonic bath (Bandelin Sonorex Digitec, DT 31) and/or a magnetic stirrer (Scansci, MS-H280Pro) were used to promote solids dissolution. Measurements of particle size distributions were made with a Mastersizer 2000 laser diffraction particle sizer (Malvern Instruments, Malvern, UK) and Mastersizer 2000 software was used for particle size analysis. The colours of the sensor materials with the eggshell were captured using a Samsung smartphone equipped with an 8-megapixel camera. The colour coordinates of each image were analysed considering the HSL colour system (hue, saturation, and brightness of HSL space). They were extracted using the Paint programme from Windows.

### 2.3. Pretreatment Eggshell

Eggshells underwent different treatments, included cleaning, drying, and grinding [12,16]. The eggshells were first cleaned in a solution having 2.0 mL sodium hypochlorite in 1.0 L of water (recommended solution for clean food/fruit/vegetables). The eggshells were then washed with water and then dried in an oven (at a temperature of 55 °C). They were then crushed, sieved through screens of various sizes, and then analysed for particle size in the Mastersizer 2000.

### 2.4. Coloured Complex

The colourimetric response was tested between OXY and metallic species, Fe(II) or Cu(II). For this purpose, 2.0 mL of the OXY solution 1.0 × 10^−3^ mol/L was mixed with 1.0 mL of an aqueous solution containing the metallic species at a concentration of 1.0 × 10^−2^ mol/L.

### 2.5. Construction of Eggshell Sensor

**Figure 1:**
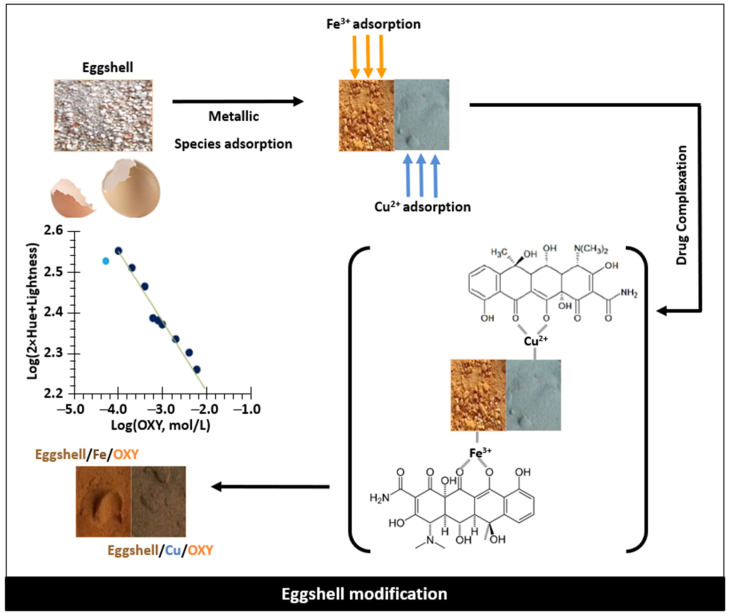
Schematic representation of the eggshell grinding and modification, starting by metal adsorption, which was followed by drug reaction to produce a coloured material that gives a linear trend between the colour coordinates and the log concentration of antibiotic.

The schematic representation of the eggshell modification to prepare the sensing material is shown in Figure 1. In detail, 0.4 g of eggshell particles were modified by incubating them in different solutions/conditions described later. Incubation was always performed with constant stirring for 3 h, usually at room temperature (~20 °C unless otherwise stated), protected from light and with eggshell submersed in the solution. After incubation, the eggshell was centrifuged, the supernatant was discarded, and the pellet was washed five times with deionized water. The clean pellet was dried in an oven at 35 °C until the eggshell was completely dry.

The chemical modification of the eggshells was performed in several steps. The first modification of eggshells was aimed at adsorption of metallic species and was attempted in several ways: (i) incubation of eggshells in a solution with metallic species prepared in deionized water; (ii) incubation of eggshells in a solution with metallic species prepared in 0.1 mol/L NaCl. Different conditions of the medium were tested to increase the porosity of the eggshells. The success of these different modifications was verified by completing the modification and incubating the modified eggshells for 5 min in aqueous solutions of OXY with different concentrations (1.0 × 10^−2^, 1.0 × 10^−3^, and 1.0 × 10^−4^ mol/L).

#### 2.5.1. Selection of Metal Concentration

Eggshells were incubated in solutions of the selected metal of 1.0 × 10^−3^, 1.0 × 10^−2^, and 1.0 × 10^−1^ mol/L with all solutions prepared in 0.1 M NaCl. The prepared eggshell sensor material (dry sensor) was then incubated in an OXY solution of 1.0 × 10^−2^ mol/L for 5 min with constant stirring. After this time, the eggshell was centrifuged, the supernatant was discarded, and the pellet was washed five times with deionized water. For the clean particles, the results were evaluated by visual comparison.

#### 2.5.2. Selection of Particle Size

The particle size of the eggshell was considered relevant for metal adsorption and was next optimized. For this purpose, the eggshell particle size obtained by pretreatment was tested (≤125 µm, 400 µm ≤ size ≤ 1400 µm, and ≥1400 µm). The OXY concentration used in this test was 1.0 × 10^−2^ mol/L.

#### 2.5.3. Adsorption Time

The time given for the adsorption of the reactants was considered relevant and optimized next. For this purpose, the time given for the reaction between the eggshell particles and the metal was varied among 1, 3, and 6 h. The OXY concentration used in this assay was 1.0 × 10^−2^ mol/L.

### 2.6. OXY Assays in the Modified Eggshell Particles

The response of the assay to OXY was checked by immersing the eggshell sensor material in a standard aqueous solution of the antibiotic for 5 min (with constant agitation). After cleaning, we allowed the particles to dry at room temperature. The image of the eggshell sensor was taken without any specific requirements and the colour coordinates were determined by analysing the image in the Paint programme. Three different points per eggshell sensor were considered and the HSL colour system was chosen to analyse the results (this approach can be replaced by more sophisticated data processing of suitable apps for reading the colour coordinates).

The procedure was applied to determine the typical calibration characteristics of the eggshell sensor, its selectivity, and its applicability in the analysis of environmental samples. Calibration was performed for aqueous solutions of antibiotics ranging from 5.0 × 10^−5^ to 1.0 × 10^−2^ mol/L. The response of the sensor was tested with other antibiotics used in aquaculture: sulfadiazine, chloramphenicol, and tetracycline. The concentration of these antibiotics was adjusted to 1.0 × 10^−2^ mol/L. The use of this sensor to quantify OXY in environmental waters was tested by spiking these waters with 3.0 × 10^−3^ mol/L, recording the colour coordinates of the incubated sensor, and calculating the corresponding concentration using the calibration data.

## 3. Results and Discussion

### 3.1. Coloured Product

Preparation of a coloured sensor material in the presence of an antibiotic is expected to cause a colour change. Since eggshell materials serve as carriers for this process, the eggshell particles must be chemically modified so that the appropriate reagents will adhere to them stably. Since OXY is the target, Fe^3+^ or Cu^2+^ was used for the reaction as published in previous studies [11]. When the concentration of OXY was adjusted to 1.0 × 10^−3^ mol/L, the concentration of the metal species was kept constant to understand the intensity of the colour formed. Overall, the blue colour of the copper species changed to a green colour in the presence of the antibiotic, while the light orange colour of the iron changed to dark brown/orange. Controls were performed to confirm that these colour changes were due to the reaction between the metal species and the antibiotic and not to changes in the substrate.

### 3.2. Construction of Eggshell Sensor

**Figure 2:**
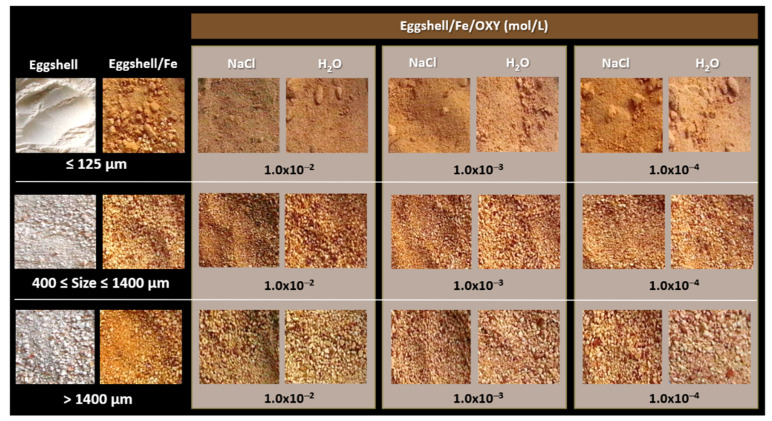
Pictures of the iron-modified eggshell materials, of different particle sizes, having previous incubation in water or NaCl, and undergoing reaction with antibiotic of concentrations ranging from 1.0 × 10^−4^ to 1.0 × 10^−6^ mol/L.

The first experiments on adsorption of the metal to eggshell particles were performed with particles of different sizes, with OXY solutions of the antibiotic prepared in water or in NaCl, and with different concentrations of the antibiotic. The results obtained for iron are shown in Figure 2. Overall, it was found that the presence of NaCl, which was used to enhance metal adsorption, resulted in more intense brown colouration. The use of NaCl impacts upon the adsorbent properties of eggshell particles, as reported previously [17]. In addition, the colour formed on the eggshell was more uniform and intense for particles with smaller dimensions. This is due to the larger surface area of the smaller particles, which increases the efficiency of adsorption.

While the modified eggshell showed a light orange colouration when in contact with an empty solution, a brown/dark orange colouration resulted when in contact with the antibiotic solution. The higher the concentration of OXY, the more intense was the brown colouration, indicating the presence of more coloured product on the particles.

### 3.3. Optimisation of the Sensor

The eggshell sensor based on adsorption of metal species was optimised for several parameters to improve colour variation between similar concentrations of OXY. An eggshell/Fe sensor was used to optimise the sensor, but eggshell/Cu performance was also tested. Results were recorded using digital photography to investigate the effects of metal concentration, particle size, and adsorption time.

#### 3.3.1. Selection of Metal Concentration

**Figure 3:**
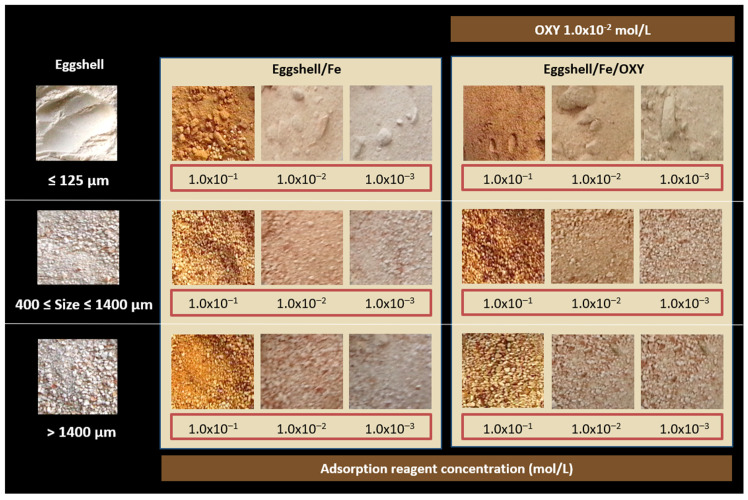
Effect of metal concentration ranging from 1.0 × 10^−3^ to 1.0 × 10^−1^ mol/L on the preparation of iron-based eggshell sensing materials having different particle sizes and a fixed concentration of antibiotic.

The concentration of the metal was tested for 1.0 × 10^−3^, 1.0 × 10^−2^, and 1.0 × 10^−1^ mol/L, and the time for this reaction was kept constant. A period of 3 h was given to ensure that the reaction took place. The concentration of the metal species is expected to affect the amount of metal bound to the eggshell particles, which in turn would dictate the amount of OXY that binds to the eggshell. The 1.0 × 10^−2^ mol/L concentration of OXY was tested for each condition, along with a blank test (in which OXY was not present). The results obtained are shown in Figure 3.

**Figure 4:**
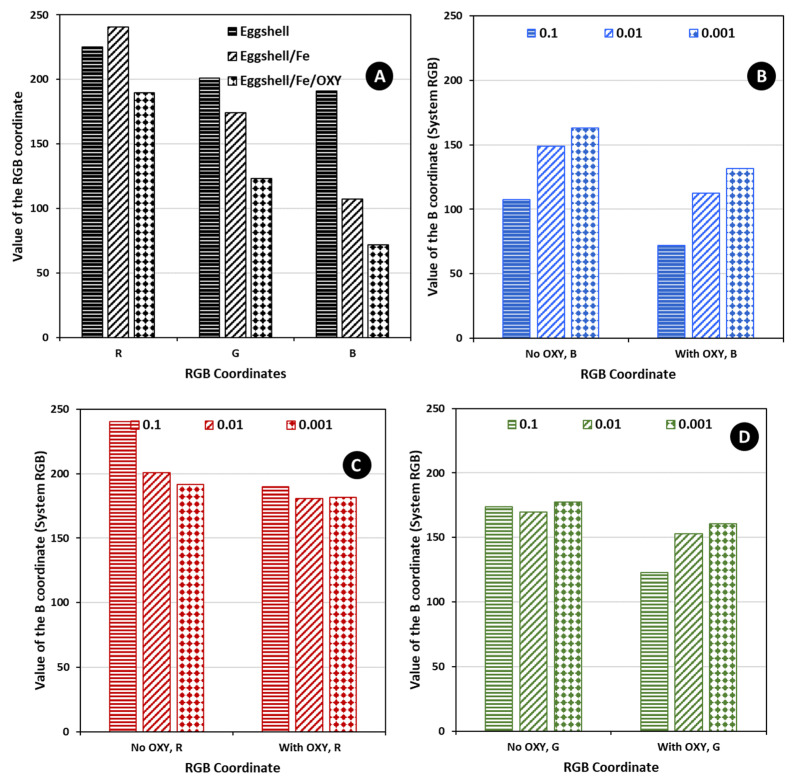
Average values of the RGB coordinates for at least 3 random measures in Paint, (**A**) for the various stages of material modification, setting the concentration of iron to 1.0 × 10^−1^ mol/L, and (**B**–**D**) for a given coordinate value obtained with the several concentrations of iron tested.

**Figure 5:**
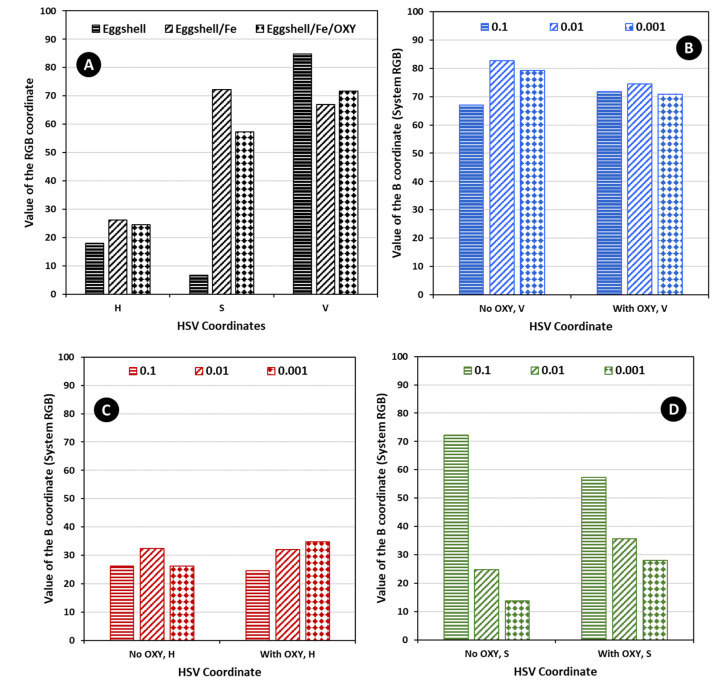
Average values of the HSV coordinates for at least 3 random measures in Paint, (**A**) for the various stages of material modification, setting the concentration of iron to 1.0 × 10^−1^ mol/L, and (**B**–**D**) for a given coordinate value obtained with the several concentrations of iron tested.

In general, the higher the concentration of the metal dissolved in the solution, the more intense the colour of the blank test. After immersing the sensors in the solutions of OXY, a significant colour change was observed (from orange to brown for the eggshell/Fe test sensor). The most significant colour changes before and after the addition of OXY were observed for the particles incubated with a solution of 1.0 × 10^−1^ mol/L iron, indicating that the concentration of the metal on their surface was higher. The observed colouration occurred almost immediately and did not change over time, up to a maximum of several months. Additional analysis of the colour coordinates was made, as shown in Figure 4 and Figure 5, plotting the RGB and the HSV coordinate values for some of the obtained data (average of at least three values). Overall, from the data obtained, it seemed that the concentration of 1.0 × 10^−1^ mol/L of iron was the one leading to the higher colour intensity changes. This was especially evident in the RGB system. Therefore, the concentration of the metal was set at 1.0 × 10^−1^ mol/L in subsequent experiments.

#### 3.3.2. Selection of Particle Size

Particle size for metal adsorption was optimised after eggshell pretreatment. Eggshells were ground and sieved with different mesh sizes. The particle sizes were determined in a granulometer and for this purpose the particle size of the pretreated eggshell was set to ≤125 µm, 400 µm ≤ size ≤ 1400 µm, and >1400 µm. As shown in Figure 2 and Figure 3, the smallest particle size promoted a more intense and uniform colour.

#### 3.3.3. Adsorption Time

**Figure 6:**
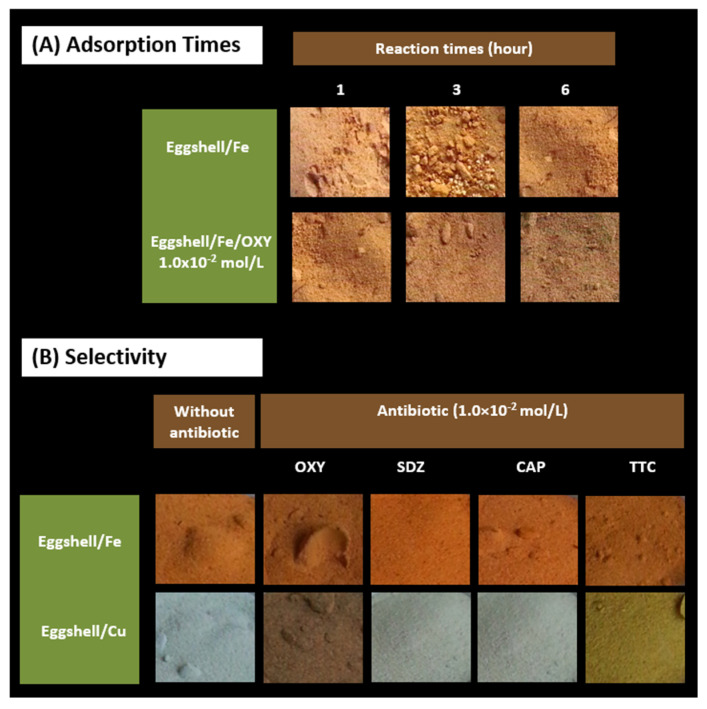
Effect of different adsorption time of the metal species to the eggshell (**A**), ranging between 1 and 6 h, and cross-response of the sensing materials (**B**) to other antibiotic drugs, set to the same concentration of the target antibiotic.

Regarding the adsorption time, it was important to determine how long the adsorption of the metal had to be promoted to produce a visible colour change when OXY was added. This variable was tested between 1 and 6 h, and the subsequent response of the sensor to an OXY solution of 1.0 × 10^−2^ mol/L was evaluated. Figure 6A summarizes the main results of this study with the eggshell/Fe sensors.

The results show that the intensity of the colour formed in the modified eggshells increased when the adsorption time was increased, although the changes observed were not very significant. The studies showed that the longer the sensor is in contact with the metallic species, the greater the colour intensity formed when incubated in an OXY solution. However, this behaviour was also observed for the eggshell background signal without OXY: the greater the amount of iron, the greater the intensity of the brown colour. Additionally, the greater difference in colour between having or not having OXY was investigated, aiming to provide a high sensitivity to the method. Therefore, an intermediate condition of 3 h of incubation was chosen to produce a greater difference between the responses of the blank solution and the standard solution OXY.

### 3.4. Cross-Response to Other Antibiotics

From an analytical point of view, the selectivity of a compound for the sensing material for which it was developed is a particularly important feature if one wishes to use that material under realistic conditions. Therefore, considering the possible use of a combination of antibiotics in aquaculture systems, the interference of the most commonly used drugs was tested. This study was performed by applying solutions of OXY, SDZ, CAP, and TTC in equal concentrations on independent optimized probes. The results obtained are shown in Figure 6B. In general, with the exception of TTC, the response of the sensor was not affected by the other drugs. This observation was independent of the metal present in the sensing surface. Since it is impractical from a therapeutic point of view to combine drugs of the same chemical category, this response would never result in true interference. Moreover, this result opened the possibility for extending the same principle to the preparation of other drugs of the tetracycline group.

### 3.5. Analytical Features of the Eggshell Sensor

The modified eggshell sensor material can be used for quantitative analyses after prior calibration against increasing concentrations of OXY. Images of the materials are then captured with a smartphone and the colour coordinates can be used to extract this information. Semiquantitative information about unknown concentrations can be obtained by visual comparison with a standard colour palette. Quantitative information can be obtained by extracting the colour coordinates using the Windows programme PAINT and mathematically processing these coordinates to obtain a suitable correlation between the concentration OXY and the colour, as described in previous work [10].

**Figure 7:**
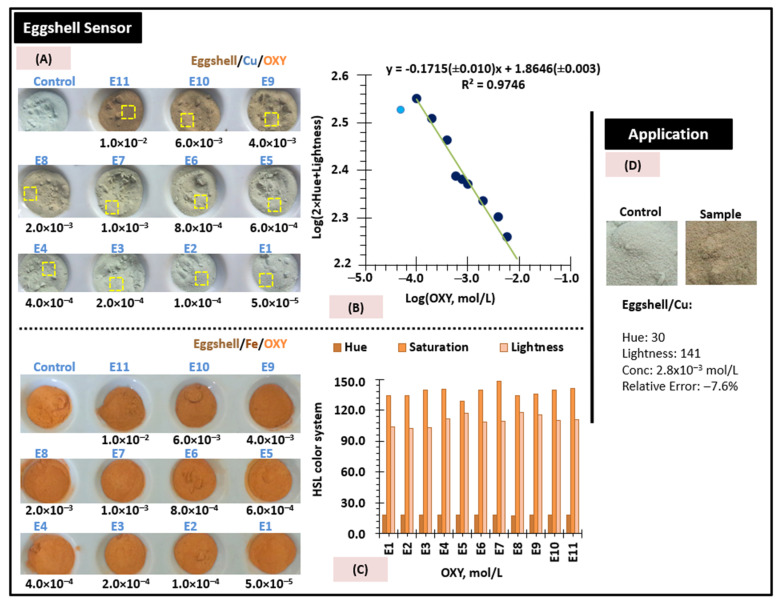
The use of the sensor, including (**A**) visual inspection and comparison with the standards; (**B**) their calibration, showing colour gradient and graphical representation of mathematical transformation of the colour coordinates with the logarithm OXY concentration for the eggshell/Cu sensor; and (**C**) evaluation of HSL colour system for the eggshell/Fe sensor, and (**D**) their application to a spiked water sample.

Iron-based sensor materials were prepared as previously described and calibrated against OXY standard solutions ranging from 5.0 × 10^−5^ to 1.0 × 10^−2^ mol/L. Each solution was incubated on the sensor materials for 5 min, and then the material was washed and dried. Unfortunately, the colours collected were not too different, which was to be expected, because the brown intensity changed only slightly (Figure 7A, bottom), although it signalled the presence of OXY. In this case, the sensor provided only a YES/NO response concerning the presence of OXY in the water solution.

Following previous experience with the detection of tetracyclines by copper and iron [11], eggshell materials were modified with copper, using the optimal procedure for iron. The eggshell/Cu sensor materials were then calibrated by incubating the modified eggshells for 5 min in OXY standard solutions ranging from 5.0 × 10^−5^ to 1.0 × 10^−2^ mol/L, as established previously for iron. The typical colours observed are shown in Figure 7A, top. The HSL coordinates were determined from the digital images and correspond to hue, saturation, and brightness, respectively. The values considered corresponded to the average of at least three experiments and were determined from the yellow square areas where homogeneous colour was observed (as shown in Figure 7A). The data were subjected to several mathematical approaches to obtain a linear trend (Figure 7B). The final data showed that Log(2 × Hue + lightness) versus Log(OXY concentration) exhibited a linear response from 1.0 × 10^−4^ mol/L, up to 1.0 × 10^−4^ mol/L.

### 3.6. Application

The use of eggshell/Cu sensor material to provide quantitative OXY data was tested in the analysis of contaminated environmental water samples. The water was environmental water spiked with OXY at a concentration of 3.0 × 10^−3^ mol/L. The colours obtained were perceptible to the human eye, and the coordinates of the digital images were acquired to estimate quantitative data from the digital image. The average H and L values were 30 and 141, as shown in Figure 7D. These values correspond to the average of three measured colour coordinates at randomly selected points. Using the linear regression equation (Figure 7B) and the coordinates given in Figure 7D, we then calculated the concentration of the sample. A concentration of 2.8 × 10^−3^ mol/L was determined for the eggshell/Cu material, corresponding to a relative error of −7.6%. Visual inspection and comparison with the standards (Figure 7A) also allowed a semiquantitative determination of the OXY amount in water.

Considering the information available in the literature, OXY has been determined by a wide range of tools, in a wide range of matrices. This has been suitably reviewed recently, in [18]. Concerning specifically test-strips, the works developed focus on environmental samples, including soil or water, in which the concentration of OXY may be rather low, much lower than the one present in aquaculture. In general, these works employ very complex and expensive designs when assembling the test-strip, also making use of compounds/materials that are not particularly environmentally friendly, such as metal organic frameworks [19] or lanthanides [20,21], in order to have changes in the emitted light when in the presence of the antibiotic. There are also whole-cell-based systems, making use of recombinant plasmids to provide a visual detection of antibiotics [22].

Overall, the present method does not have a better analytical performance than the previously reported methods, because it employs a simple redox reaction to undergo a colourimetric reaction with OXY and allows visual detection or use of smartphone to provide semiquantitative information. However, the use of eggshell/Cu material proved to be simple and accurate for the intended purpose, as results were obtained simply by incubating a modified eggshell in the solution to be analysed. Further use of eggshells/Fe in an array format is expected, as the iron-based responses confirm that the response results from the presence of OXY. This approach increases the significance of the analytical data generated.

## 4. Conclusions

For rapid OXY screening in the aquatic environment, a low-cost disposable optical probe was developed specifically for monitoring antibiotic concentrations introduced into the aquatic environment. The use of eggshell material as substrate gives this approach an especially important sustainability feature. Eggshell is a waste from a natural product of human use, and it may be reused as a simple substrate for colour measurements in test-strips.

The chemical modification of eggshell by adsorption of metal species (Cu^2+^ or Fe^3+^) enabled the formation of colour changes on the surface that could be quickly and easily detected by the naked eye. The eggshell modified with copper was more sensitive to lower concentrations than the eggshell modified with iron. The resulting colour intensity, observed with a digital camera, was directly proportional to the logarithm of the concentration OXY, allowing more accurate quantification. The probe showed high selectivity for the target analyte.

The response of the material to other tetracycline antibiotics allowed detection of the tetracycline group, indicating that this material can be used for monitoring other tetracyclines and its response is not affected by other drugs that may be present in the medium. While the performance compared to other test-strips is poorer, its cost-effectiveness and sustainability is much greater. The method reported herein uses a simple material containing eggshell residues and adsorbed metal species, which may also provide advantages in terms of circularity of the used materials and the reduced impact of the generated wastes. The sustainability of the procedure is expected to be great, when considering the possibility of giving a second life to eggshell wastes, as there is no relevant use for this material so far.

Overall, the eggshell sensors proposed here are inexpensive, enable rapid, low-cost, simple, instrument-free, and environmentally friendly determinations, and are therefore suitable for field applications. They can be obtained in large quantities from restaurants and confectioneries or even private households, provided that selective collection is performed.

## Data Availability

Data is contained within the article.
